# Ketone bodies determine the female reproductive lifespan

**DOI:** 10.1093/lifemeta/loac018

**Published:** 2022-08-18

**Authors:** Long Yan, Hongmei Wang

**Affiliations:** State Key Laboratory of Stem Cell and Reproductive Biology, Institute of Zoology, Chinese Academy of Sciences, Beijing 100101, China; Institute for Stem Cell and Regeneration, Chinese Academy of Sciences, Beijing 100101, China; Beijing Institute for Stem Cell and Regenerative Medicine, Beijing 100101, China; University of Chinese Academy of Sciences, Beijing 100049, China; State Key Laboratory of Stem Cell and Reproductive Biology, Institute of Zoology, Chinese Academy of Sciences, Beijing 100101, China; Institute for Stem Cell and Regeneration, Chinese Academy of Sciences, Beijing 100101, China; Beijing Institute for Stem Cell and Regenerative Medicine, Beijing 100101, China; University of Chinese Academy of Sciences, Beijing 100049, China

**In a recent study published in *Life Metabolism*, Wang and colleagues reported that the ketone body was elevated during the neonatal stage to regulate the formation of the primordial follicle pool and affect the subsequent ovarian aging through coordinating ROS-induced follicle apoptosis.**

Female reproduction and health are highly dependent on ovarian function. The critical indicator of ovarian function is the ovarian reserve [1], which refers to the stockpile of dormant primordial follicles (primordial follicle pool) residing in the ovarian cortex [2]. The initial size of the primordial follicle pool and the rate of primordial follicle consumption determine the extent and the speed of reproductive aging [3]. Extensive studies have reported multiple mechanisms whereby the formation of primordial follicle pool is regulated [4]. Furthermore, increasing evidence reveals that environmental chemicals contribute to the ovarian reservoir and the reproductive lifespan [5]. However, the association between surroundings and ovarian activities remains poorly understood.

Ketone bodies are vital alternative metabolic fuel source for all the domains of life [6]. In mammals, ketone bodies are produced by the liver and used peripherally as an energy source when glucose is not readily available. The ketone bodies refer to three molecules, acetoacetate (AcAc), 3-β-hydroxybutyrate (β-HB), and acetone. AcAc and β-HB are predominant and energy-rich compounds which transport energy from liver to essentially all of the body tissues for terminal oxidation [7]. Particularly, β-HB functions to orchestrate the antioxidant defence program and maintain redox homeostasis in response to environmental and metabolic challenges [8]. It was reported that oxidation of ketone bodies may disturb sperm motility and lead to male infertility [9], and ketone bodies are also found in the blood of neonates and pregnant women. It is interesting to study if the ketone bodies affect ovarian reserve.

A recent study found that the colostrum-activated ketone body at the postnatal stage regulated the size of the primordial follicle reservoir and affected ovarian aging via reducing the oxidative stress and damage [10]. First, the authors studied the expression pattern of the ketone body and its catalytic enzyme Hmgcs2 during the period of primordial follicle pool formation. They found the levels of ketone bodies and Hmgcs2 were significantly increased from embryonic to neonatal stages. Deletion of *Hmgcs2* resulted in smaller ovarian follicle reservoir as manifested by decreased litter size and prolonged litter interval with age increased. The above results indicated the ketone body regulated the formation of the ovarian follicle pool during neonatal stage and was related to the maintenance of the ovarian function in adulthood. Next, to determine the intrinsic mechanism of ketone body deficiency to the depleted ovarian follicle pool formation, the authors performed single-cell RNA-seq using neonatal ovaries from wild type and *Hmgcs2* deletion mice. They found that deficiency of ketone body did not affect the cell lineage differentiation, but the ROS pathway was largely enhanced in endothelial, epithelial, granulosa, and stromal cells. As the pregranulosa cells are vital for oocyte develop and germ cyst breakdown [11, 12], the authors then specifically knocked out *Hmgcs2* in the granulosa cells and found no impact in the ovarian morphology and follicle development, which suggested that it was the systemic *Hmgcs2* but not the granulosa cell-specific *Hmgcs2* that affected the ovarian reserve. Finally, the authors investigated the mechanism by which the ketone body manipulated the ovarian reserve. They found the elevated ROS level according to the destroyed ketone body aggravated DNA damage, which further augmented follicle atresia, and ultimately led to the severe reduction of the primordial follicle pool. β-HB supplementation saved primordial follicle pool defects and reduced ROS production in the ovary of ketone body deficient neonatal mice. To summarize, these experiments elucidate that neonatal ketone body defect results in the elevation of ROS level and excessive apoptosis which together could decrease the initial ovarian reserve and shorten the female reproductive lifespan.

Overall, this study provides a picture outlining adverse nutrition conditions in foetal and infant stages disturbing follicle production and leading to a limited primordial follicle pool and reproduction. Our proposals for future investigations are: (i) How does the environmental input exactly affect the establishment and dynamics of the ovarian reserve? What is the pivotal time point? (ii) In addition to functioning as alternative energy source during energy stress, it has been well established that β-HB also moonlights as a signalling molecule [8]. Herein, how do the hormones and nutrient signaling coordinate to involve in this program? (iii) As increasing factors or conditions have been identified to affect the ovarian reserve [13], more experimental investigations and epidemiological surveys on humans or alternative primates are needed to determine their effects on the follicle reservoir and ovarian functions, which will definitely help to develop new medical treatments to expand the ovarian reserve, prolong the reproductive lifespan and postpone the menopause.
